# Quantum dots – a versatile tool in plant science?

**DOI:** 10.1186/1477-3155-4-5

**Published:** 2006-06-15

**Authors:** Frank Müller, Andreas Houben, Peter E Barker, Yan Xiao, Josef A Käs, Michael Melzer

**Affiliations:** 1 Institute of Plant Genetics and Crop Plant Research (IPK), 06466 Gatersleben, Germany; 2 University of Leipzig, Faculty of Physics and Geosciences, Leipzig, Germany; 3 National Institute of Standards and Technology, Gaithersburg, Maryland 20899, USA

## Abstract

An optically stable, novel class of fluorophores (quantum dots) for *in situ *hybridisation analysis was tested to investigate their signal stability and intensity in plant chromosome analyses. Detection of hybridisation sites *in situ *was based on fluorescence from streptavidin-linked inorganic crystals of cadmium selenide. Comparison of quantum dots (QDs) with conventional detection systems (Alexa 488) in immunolabeling experiments demonstrated greater sensitivity than the conventional system. In contrast, detection of QDs in *in situ *hybridisation of several plant chromosomes, using several high-copy sequences, was less sensitve than Alexa 488. Thus, semiconductor nanocrystal fluorophores are more suitable for immunostaining but not for *in situ *hybridisation of plant chromosomes.

## Background

Quantum dots (QDs) have been introduced as a promising new tool in life sciences, because of their unique optical properties [1]. They are highly stable during excitation and have characteristic absorption and emission spectra [2]. The emission peak of these nanoparticles is comparatively narrow and the dots fluoresce brighter than organic fluorescent dyes. Thus particle visibility is enhanced and weaker laser intensity is required for the imaging process. QDs can be excited using different wavelengths from UV up to the emission wavelength. Hence it is possible to excite simultaneously QDs emitting at different wavelengths potentially facilitating a simpler handling of multicolor-labelled samples.

Initial attempts to synthesize semiconductor crystals resulted in QDs trapped in glass [2-4]. Later, nanoparticles were developed that could be dispersed in various solvents and whose surface could be derivatized. After hydrophilic coating of QDs with mercaptoacidic acid, dehydrolipoic acid or other reagents, nanocrystals became applicable in biology [5]. The core of the quantum dot particle is composed of a mixture of cadmium and selenide. This sphere, having a diameter of 20 to 55 Å [6], is coated with 1–2 monolayers of ZnS measuring 3,1 Å.

Proteins, antibodies, DNA or other molecules of interest can be attached to QDs allowing a wide range of applications in life sciences [7]. The complete QD-streptavidin conjugate has a diameter of 10 to 15 nm [8]. Hence quantum dots have been employed in live cell imaging [1,9], diagnostic and therapeutic purposes [10], immunohistochemistry [11] and in fluorescence *in situ *hybridisation (FISH) experiments [12,13]. However, until now there have been no reports of applications of QDs in plant research [14,15]. In order to test whether the application of nanoparticle techniques could improve the sensitivity of *in situ *hybridisation on plant chromosomes, we conducted a range of comparative test experiments. In addition QDs were employed for immunolabelling of tissue sections.

## Materials and methods

### Material

For *in situ *hybridisation of young seedlings of *Allium fistulosum samples *were pre-treated in iced water for 24 h, fixed in ethanol-glacial acetic acid (3:1, v/v) for 2 days at 4°C and stored at 4°C in 70% ethanol. The ethanol/acetic acid-fixed material was prepared as described in [16]. Alternatively, root meristems were fixed for 30 min in freshly prepared 4% (w/v) formaldehyde solution containing phosphate-buffered saline (PBS, pH 7.3), washed for 45 min in PBS and digested at 37°C for 25 min in a mixture of 2.5% pectinase, 2.5% cellulase Onozuka R-10 and 2.5% pectolyase Y-23 (w/v) dissolved in PBS prior to slide preparation.

### Fluorescence *in situ *hybridisation (FISH)

The generation of probes specific for the *A. fistulosum *non-coding satellite sequence [17] was performed as described by [18]. A plasmid VER17 [19] encoding part of the 18S, the 5.8S, most of the 25S and the internal transcribed spacers of *Vicia faba *45S rRNA, was used as a rDNA-specific probe.

*In situ *hybridisation probes were labelled by nick translation with digoxigenin-11-dUTP or biotin-16-dUTP. FISH was carried out according to [20]. For combined probing of rDNA and non-coding satellite DNA, *in situ *hybridisation was performed using 20 ng of digoxigenin-labeled 45S rDNA and 20 ng of biotin-labelled satellite DNA per slide. Hybridisation sites of the digoxigenin- or biotin-labelled probes were detected using the conventional detection systems, anti-digoxigenin-rhodamine antibody, or streptavidin-Alexa 488 respectively, each at a concentration of 2 μg/ml. In parallel, hybridisation sites of the biotin-labelled probe were detected by using 20 pM QD 565 streptavidin conjugate (Quantum Dot Corporation, USA). The incubation times were 1 h for Alexa 488 and Rhodamine, each, and 2 h for QD565. Working solutions of QDs and antibodies were prepared either in 4 × SSC,1% BSA, 0.1% Tween 20 or Borate buffer (50 mM boric acid H_3_BO_3_, pH 6.0 or 7.0). After final washing steps and dehydration, the tissues were mounted in antifade medium containing 10μg/ml DAPI. Fluorescence signals were recorded electronically with a confocal Laser-Scanning-Microscope LSM 510 META (Carl Zeiss Jena GmbH, Jena, Germany) by using laser line 488 for Alexa 488, 543 for rhodamine and 364 for DAPI and QD 565 excitation. Additionally a cooled CCD-camera attached to a standard fluorescence microscope (BX61, Olympus) was used The image manipulations were performed with the program Adobe Photoshop.

### Immunolabelling of sectioned material

One mm^2 ^leaf sections of *Zea mays *were fixed for 3 h at room temperature in 50 mM cacodylate buffer (pH 7.2), containing 0.5% (v/v) glutaraldehyde and 2.0% (v/v) formaldehyde after short vacuum-infiltration. After the fixation the samples were dehydrated in stepwise fashion by adding progressively increasing concentrations of ethanol and concomitantly lowering the temperature (PLT) using an automated freeze substitution unit (AFS, Leica, Benzheim, Germany). The steps used were as follows: 30% (v/v), 40% (v/v) and 50% (v/v) ethanol for 1 h each at 4°C; 60% (v/v) and 75% (v/v) ethanol for 1 h each at -15°C; 90% (v/v) ethanol and two times 100% (v/v) ethanol for 1 h each at -35°C. The samples were subsequently infiltrated with Lowycryl HM20 resin (Plano GmbH, Marburg, Germany) by incubating them in the following mixtures: 33% (v/v), 50% (v/v) and 66% (v/v) HM 20 resin in ethanol for 4 h each and then 100% (v/v) HM 20 overnight. Samples were transferred into gelatine capsules, incubated for 3 h in fresh resin and polymerized at 35°C for 3 days under indirect UV light. 0.5 μm thick sections of the embedded plant tissue were cut with a diamond knife using an Ultramicrotome (Leica Microsystems AG, Wetzlar, Germany) and mounted on slides at 60°C. These sections were washed for 3 × 5 minutes in PBS + 1% BSA at room temperature (RT) and blocked for 20 minutes in PBS + 3% BSA at RT. An antibody from rabbit against CF1 (catalytic portion of the chloroplast H^+^-ATP synthase), against chloroplasts [21], and afterwards an anti- rabbit IgG- Biotin conjugate (30 min, both diluted in PBS + 1% BSA, RT) was attached. The secondary antibody was detected with the quantum dot 565-streptavidin conjugate. Each step except blocking was followed by washing as previously described.

### Transmission electron microscopy

An aliquot of the QD 565 streptavidin conjugate at a concentration of 0.2 nM was pipetted onto a formvar-coated grid. The grids were pre-treated with poly-L-lysine to increase the binding of the particles. After one minute the grid was drained onto paper and one droplet of 4% uranyl acetate was added. After 15 s the draining procedure was repeated and the grids were air dried. Images were recorded using a Zeiss EM 902 A electron microscope (Carl-Zeiss GmbH, Oberkochen, Germany), equipped with a Megaview III CCD camera (Soft Imaging System, Münster, Germany).

## Results and discussion

**Figure 1:**
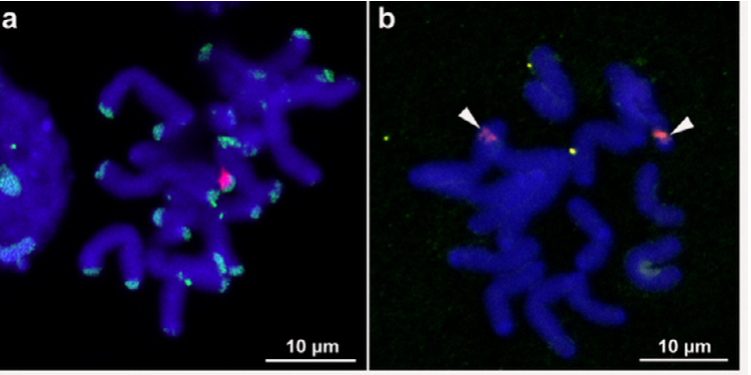
(a) Somatic chromosome and nuclei of *Allium fistulosum *after DAPI staining and fluorescence *in situ *hybridization with labelled non-coding satellite sequences (green signals for Alexa 488) and 45S rDNA (red signals for rhodamine) using conventional detection systems. (b) *A. fistulosum *chromosomes after *in situ *hybridisation with the same probes. The biotinylated non-coding satellite probe was detected with QD 565 streptavidin conjugate (green signal, arrowed) yielding only very weak and few signals. In contrast, the conventional antibody detection of 45s rDNA loci (in red) resulted in a strong hybridization signal.

To test whether the unique optical properties of QDs could be used to improve the signal intensity and stability of *in situ *hybridised probes, chromosomes were hybridised with different types of high copy sequences. Using the conventional detection system (Alexa 488, rhodamine), strong hybridisation signals of the biotin-labelled *A. fistulosum *satellite or digoxigenin-labelled 45S rDNA were detectable at the expected chromosomal sites (Fig. 1a). After confirming the suitability of the probes and of the hybridisation procedure, the same conditions were used to test the suitability of quantum dot technology for detection of *in situ *hybridised probes. Therefore, instead of detecting the biotinylated probe by streptavidin-Alexa 488 we employed a QD 565-streptavidin conjugate. However, when visualised by both types of fluorescence microscopes, QDs revealed only very weak hybridisation signals, while the anti-digoxigenin-rhodamine detected 45S rDNA control signals were always clearly visible (Fig. 1b).

**Figure 2:**
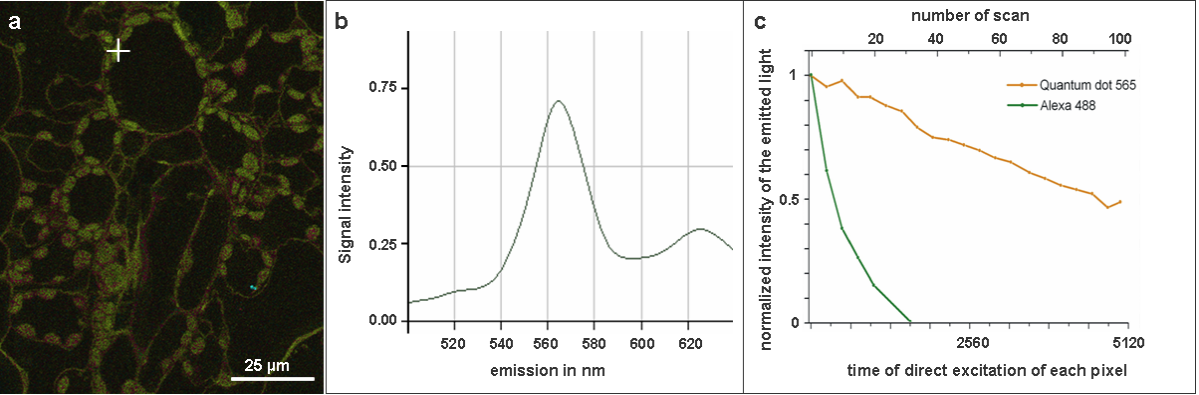
(a) Immunolabelled section of a resin-embedded *Zea mays *leaf using a CF1- antibody and subsequent signal detection by a combination of an anti-rabbit IgG- Biotin/quantum dot 565-streptavidin conjugate. Note the strongly labelled chloroplasts. White cross indicates position of the signal used for determination of the fluorescence spectrum. b) The graph shows the emission wavelengths versus their intensity in arbitrary units. (c) Comparison of stability of QD 565 versus Alexa 488 signals indicates a high stability of QD-based signals. Leaf sections immunolabelled with CF1-antibody detected by QD 565-streptavidin conjugate or Alexa 488-streptavidin conjugate and laser scanned 100 times.

To monitor the quality of the QD conjugate employed for the *in situ *hybridisation experiments, the same QDs were used for immunolabelling of leaf sections of *Zea mays *with the anti-CF1 antibody. Confocal laser scanning microscopy imaging revealed a strong and CF1-specific immunolabelling of chloroplasts (Fig. 2a). The emission spectrum peaks at 565 nm, hence demonstrating the functionality of the QDs tested for immunohistochemistry (Fig 2b). In parallel, anti-CF1 signals were detected using an Alexa 488 streptavidin conjugate as control. To compare the stability of Alexa 488 and QD 565-signals, both probes were laser scanned repeatedly 100 times. Immunofluorescence of nanocrystal fluorophores was significantly brighter and more photostable (Fig. 2c) than the organic fluorophore Alexa 488, as previously demonstrated in similar applications [2].

To improve the performance of quantum dots in *in situ *hybridisation the following strategies were tested: (1) instead of fixation in an ethanol : acetic acid solution, plant material was fixed in freshly prepared 4% parafomaldehyde for 25 min; (2) to increase the accessibility of chromosomes, different pepsin treatments were used and nuclei were prepared without cytoplasm and (3) 50 mM borate buffer (at pH 6.0 or pH 7.0) was used instead of 2 × SSC. In addition, (4) the concentration of the QD working solution was increased up to ten-fold which resulted in strong background fluorescence (data not shown). (5) Hybridisation of plant chromosomes using the same conditions as those published for mammalian chromosomes using quantum dot-based detection of *in situ *hybridised probes [13] were also tried out. Although a number of different possibilities were tested, none of these changes resulted in significantly improved quantum dot-based *in situ *hybridisation signals in plants. Further, no improvement in *in situ *hybridisation site detection was obtained with a QD 605 streptavidin conjugate or by using a rabbit anti-biotin antibody detected by a QD 565 anti-Rabbit IgG conjugate (both: Quantum Dot Corporation, USA). Additionally, similar results were obtained for detection of labeled 45S rDNA on chromosomes of *Arabidopsis thaliana *and *Nicotiana tabacum *using quantum dots.

**Figure 3:**
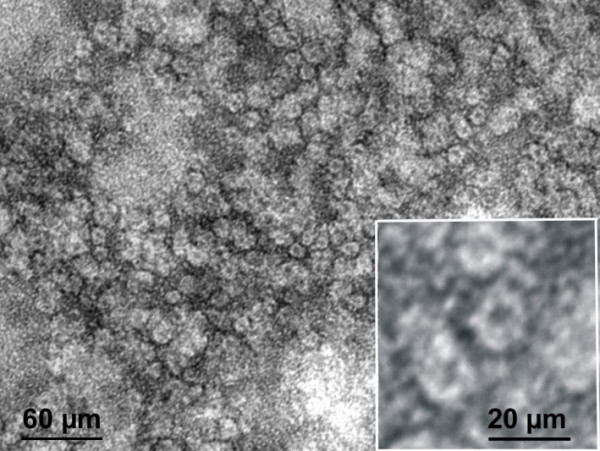
Transmission electron micrograph of the QD 565 streptavidin conjugate after negative staining reveals a particle size of 15 nm. Further enlarged quantum dots are shown in square inset.

Why was the signal detection of *in situ *hybridised probes via quantum dots comparatively lower on the chromosomes of plants when the application of this technique to mammalian chromosomes was efficient? We suspect that lack of labels on chromosomes could be due to sterical hinderance of the rather large quantum dots into the more densely packed plant chromatin, compared to animal chromatin [22]. Further, the formamide treatment required for *in situ *hybridisation of chromosomes causes considerable changes in the chromatin structure [23], which could negatively influence the accessibility of chromatin. Measurement of the size of the quantum dots revealed a diameter of 15 nm per dot (Fig. 3), whereas that of Alexa 488-streptavidin is only 0.6 nm, suggesting a much greater capability to penetrate chromatin. Notably, a size dependence on the accessibility of immunoreactants in fixed chromatin was discussed for immunogold markers [24]. These results suggested that, for sterical reasons, the immunolabelling of plant chromosomes could be performed with 1.4 nm Nanogold-labelled antibodies, but not with 10 nm gold-labelled antibodies.

In summary, while quantum dot-based immunodetection is a promising new tool in plant science, it seems that problems of handling the nanocrystals occur in FISH experiments with plant chromosomes. We suggest that these large semiconductor nanocrystal fluorophores suffer from steric hinderances which preclude their use in *in situ *hybridisation to plant chromatin.
